# Beyond ‘One Size Fits All’: Abatement of Linezolid‐Induced Thrombocytopenia Through Therapeutic Drug Monitoring and Precision Dosing—A Case Study

**DOI:** 10.1155/crdi/3214119

**Published:** 2026-04-19

**Authors:** Kennedy C. Concannon, Taylor Gullickson, Daniel C. DeSimone, Omar Abu Saleh, Tanner M. Johnson, Ryan W. Stevens, Josh Clement

**Affiliations:** ^1^ Department of Pharmacy, Mayo Clinic, Rochester, Minnesota, USA, mayo.edu; ^2^ Department of Pharmacy, University of North Carolina Medical Center, Chapel Hill, North Carolina, USA; ^3^ Division of Public Health, Infectious Diseases, and Occupational Medicine, Mayo Clinic, Rochester, Minnesota, USA, mayo.edu; ^4^ Department of Cardiovascular Diseases, Mayo Clinic, Rochester, Minnesota, USA, mayo.edu; ^5^ Department of Pharmacy, NewYork-Presbyterian/Columbia University Irving Medical Center, New York City, New York, USA

**Keywords:** chronic suppression therapy, individualized dosing, linezolid-induced thrombocytopenia, recurrent bacteremia, therapeutic drug monitoring

## Abstract

**Background:**

Linezolid is an oxazolidinone antibiotic used in the treatment of Gram‐positive bacterial infections. However, its prolonged use is often limited by adverse effects, including thrombocytopenia. Therapeutic drug monitoring (TDM) has been proposed to individualize linezolid dosing to mitigate such adverse effects. This study presents a case of linezolid‐induced thrombocytopenia successfully managed with TDM and dose individualization in a patient receiving linezolid as chronic suppressive therapy for cardiac implantable electronic device (CIED) infection.

**Methods:**

A 92‐year‐old female with a history of recurrent *Corynebacterium striatum* bacteremia and complex cardiovascular conditions who completed a six‐week course of intravenous therapy and were placed on chronic suppressive therapy with oral linezolid. Given the risk of thrombocytopenia, TDM was utilized to guide linezolid dosing. Platelet counts and linezolid trough levels were regularly monitored, and dose adjustments were made accordingly.

**Results:**

Upon initiation of linezolid (600 mg daily), the patient’s baseline platelet count was 150 × 10^9^/L. On day 19, platelet counts declined to 73 × 10^9^/L. Linezolid was temporarily withheld, and TDM revealed a supratherapeutic trough level of 10.26 mcg/mL. The dosage was adjusted to 300 mg once daily, leading to the stabilization of platelet counts at 140 × 10^9^/L. Subsequent monitoring showed a trough level within the target range (7.62 mcg/mL) with no further episodes of thrombocytopenia or additional adverse effects out to day 172.

**Conclusion:**

This case highlights the utility of TDM in managing linezolid‐induced thrombocytopenia, particularly in patients requiring prolonged therapy. TDM enabled precise dose adjustments, ensuring therapeutic efficacy while minimizing toxicity. The findings support the broader implementation of TDM in linezolid therapy to enhance patient safety and treatment outcomes, particularly in long‐term therapy or deep‐seated infections.

## 1. Introduction

Linezolid is an oxazolidinone antibiotic that inhibits protein synthesis by binding the P‐site of the ribosomal 50s subunit [1]. It is available as both oral and intravenous (IV) formulations, is 100% bioavailable when given orally, and provides broad Gram‐positive activity, thereby making it an attractive option for both inpatient and outpatient antibiotic therapy. However, the potential for adverse reactions, such as lactic acidosis, peripheral neuropathy, and thrombocytopenia, can limit its utilization in practice [2].

Thrombocytopenia is a common adverse effect leading to linezolid discontinuation. The risk of thrombocytopenia increases after 2 weeks of linezolid therapy, which is particularly concerning for infections requiring long durations of therapy, such as osteomyelitis or infective endocarditis, or for use as chronic suppression. While the package insert recommends a maximum duration of therapy of 28 days, there are clinical scenarios where extended use is necessary when other antimicrobial options are limited. Therapeutic drug monitoring (TDM) has been proposed as a strategy to individualize linezolid dosing and circumvent the development of thrombocytopenia. Herein, the authors present a case of linezolid‐induced thrombocytopenia that was successfully managed with TDM‐guided dose modification in a patient undergoing chronic suppression therapy with linezolid following recurrent *Corynebacterium striatum* bacteremia with retained hardware.

## 2. Case Report

A 92‐year‐old female was transferred to our medical intensive care unit for the management of cardiogenic and septic shock. Pertinent past medical history included rheumatic heart disease status post tricuspid valve annuloplasty, bioprosthetic aortic valve replacement, valve‐in‐valve mitral valve replacement, coronary artery disease status post coronary artery bypass surgery, tachycardia‐bradycardia syndrome, and nonsustained ventricular tachycardia status post single‐chamber pacemaker and implantable cardioverter‐defibrillator placement. The patient presented to an outside facility one day prior to transfer with symptoms of asthenia, dizziness, productive cough, and multiple episodes of non‐bloody emesis and loose stools over the preceding 2 days. Evaluation in the emergency department revealed mild pulmonary edema and increased left basilar airspace opacities suggestive of pneumonia versus atelectasis. Upon admission, she was normotensive with a blood pressure of 130/71 mmHg, tachycardic with a heart rate of 128 beats per minute, tachypneic with a respiratory rate of 32 breaths per minute, and febrile with a temperature of 39.9°C. Laboratory findings revealed a normal leukocyte count of 8.8 × 10^9^ cells/L (reference range, 3.4–9.6 × 10^9^ cells/L) and an elevated lactate level of 3.3 mmol/L (reference range, 0.5–2.2 mmol/L). Shortly after admission, she became hypotensive with a blood pressure of 71/45 mmHg. Blood cultures were collected, IV fluid resuscitation was initiated, and she was started on ceftriaxone and azithromycin for suspected community‐acquired pneumonia. Her lactate continued to rise, and she remained in refractory shock, requiring vasopressors.

On hospital day two, blood cultures were repeated, and the patient’s antimicrobial regimen was escalated to vancomycin and cefepime with subsequent clinical stabilization. On hospital day three, infectious diseases (ID) was consulted after 3 of 3 sets of blood cultures from the outside facility yielded *Corynebacterium striatum/simulans* after 14 h. Consequently, cefepime was discontinued, and vancomycin monotherapy was maintained pending antimicrobial susceptibility testing (AST). There was significant concern for CIED infection and/or prosthetic valve endocarditis; however, neither transthoracic nor transesophageal echocardiogram demonstrated evidence of valvular endocarditis or discrete vegetations on CIED leads. Blood cultures from hospital days two, three, and four remained negative. Considering the patient’s significant comorbidity burden, prolonged duration of CIED placement (20 years), and documented microbiologic clearance on repeat blood cultures, she was deemed ineligible for CIED extraction. On hospital day seven, AST, via agar dilution, of the *C. striatum* isolate demonstrated susceptibility to vancomycin but resistance to ceftriaxone, meropenem, and penicillin. Vancomycin was continued to complete a 14‐day course and the patient was discharged to a skilled nursing facility on hospital day 15. At outpatient ID clinic follow‐up 7 days after discharge, she was doing well and repeat blood cultures drawn at that visit were negative.

Five days after outpatient ID follow‐up, she presented to her local emergency department with concerns of fever (temperature of 39.1°C), nausea/vomiting, decreased enteral nutrition, weakness, and dizziness. Blood cultures were collected, empiric vancomycin was initiated, and the patient was transferred to our facility. Upon transfer, her blood cultures were growing Gram‐positive bacilli after 17 h of incubation. On hospital day three, her blood cultures were again identified as *C. striatum*, and expanded AST revealed susceptibility to vancomycin, daptomycin, and linezolid but resistance to penicillin, ceftriaxone, meropenem, ciprofloxacin, doxycycline, tetracycline, and sulfamethoxazole/trimethoprim. Blood cultures collected on hospital day three were again positive for *C. striatum* after 18 h. Blood cultures from hospital day six would remain negative after 5 days of incubation. A repeat transesophageal echocardiogram did not show any cardiac valve or lead vegetations. However, CIED infection was the most likely etiology of the patient’s recurrent bacteremia. The patient remained ineligible for device extraction and was discharged home on hospital day 11 to complete a 6‐week course of outpatient parenteral vancomycin with a referral to the ID clinic for follow‐up upon completion of this regimen.

**FIGURE 1:**
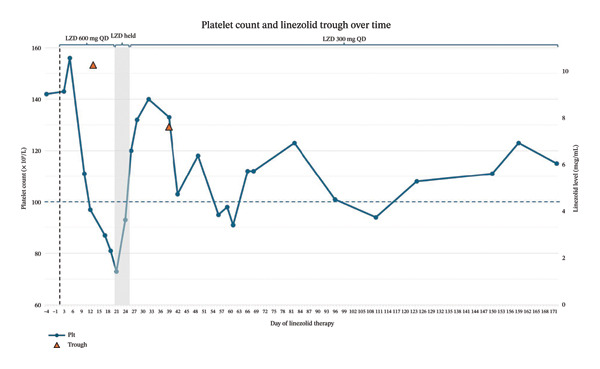
Platelet count and linezolid trough concentrations over course of therapy. Blue line indicates platelet count and orange line indicates trough levels. Abbreviations: LZD, linezolid; QD, once daily; Plt, platelets.

At the time of ID follow‐up and in accordance with the patient’s goals of care, initiation of chronic suppression therapy was pursued. Linezolid represented the only oral antimicrobial agent to which the *C. striatum* was susceptible. Given the patient’s risk factors for linezolid‐related toxicities, including age (92 y/o), weight (55 kg), and baseline renal impairment (eGFR 50 mL/min), the dose was empirically reduced to 600 mg orally once daily with plans to obtain TDM and twice‐weekly complete blood counts. On initiation of linezolid therapy, the patient’s baseline platelet count was recorded at 150 × 10^9^/L (Figure 1). During her follow‐up appointment with the outpatient ID clinic on day 13 of therapy, the patient reported tolerating linezolid well, though her platelet count had decreased to 97 × 10^9^/L. A linezolid trough (24 h after her previous dose) was also collected at this time. The patient’s platelet count continued to decline, dropping to a nadir of 73 × 10^9^/L on day 21 of therapy. Consequently, linezolid was withheld from days 20–24. The linezolid trough returned on day 24 and resulted as supratherapeutic at 10.26 mcg/mL (goal: 2–8 mcg/mL) [1]. Linezolid was reintroduced on day 25 at a reduced dose of 300 mg orally once daily. This adjustment led to an improvement in platelet count, which returned to 140 × 10^9^/L within a week. Repeat linezolid trough on day 39 of therapy resulted at 7.62 mcg/mL. No further TDM was pursued as the patient’s platelet count has stabilized and remained near baseline out to day 172 of therapy on a dose of 300 mg PO daily.

## 3. Discussion

Despite the lack of formal dose adjustment recommendations for renal or hepatic impairment, standard 600 mg twice daily dosing results in widely varying serum concentrations, with supratherapeutic concentrations associated with the development of toxicities, such as thrombocytopenia [3, 4]. One 10‐year retrospective study identified those standard dosing produced concentrations between 2 and 7 mcg/mL in 50.8% of cases, with 33% displaying supratherapeutic and 16.2% displaying subtherapeutic serum concentrations [3]. In line with these findings, our case demonstrates the critical role of pre‐emptive TDM in managing linezolid‐induced thrombocytopenia, particularly in patients presenting with multiple predisposing risk factors and in the context of long‐term therapy. Our patient, with a marginal baseline platelet count of 150 × 10^9^/L, advanced age, small body habitus, and underlying renal insufficiency, possessed multiple risk factors for linezolid‐induced thrombocytopenia. Advanced age may further contribute to altered linezolid exposure through age‐related reductions in hepatic metabolic capacity, decreased hepatic blood flow, and changes in body composition that influence drug distribution and clearance. Although formal dosing adjustments based on age alone are not currently recommended, these physiological changes in elderly patients may predispose to drug accumulation and toxicity. These risk factors, combined with the need for prolonged duration of therapy, underscored the necessity of precision dosing using TDM [1, 5]. Multiple ranges for therapeutic serum concentrations have been suggested and evaluated in linezolid TDM literature, most suggesting the maintenance of a trough concentration between 2 and 8 mcg/mL, consistent with what was used in our case [1, 6–8]. Avoidance of supratherapeutic serum concentrations correlated with the resolution of thrombocytopenia, facilitating use of a total daily dose one‐quarter of that recommended by the manufacturer without recurrence of infection out to day 172 [1, 2].

The importance of TDM in mitigating linezolid‐induced adverse effects is further substantiated by both prospective and retrospective studies. In a retrospective cohort study conducted by Lau et al., linezolid‐induced thrombocytopenia was identified as a principal factor in the premature discontinuation of linezolid, accounting for early cessation in 58% of affected patients [9]. In this study, 23% of patients underwent TDM with dose individualization, which notably reduced the likelihood of toxicities (aOR = 0.45, 95% CI 0.21–0.96, *p* = 0.038).

A prospective, open‐label study by Cojutti et al. found that proactive TDM in patients treated with linezolid for more than 10 days enabled correction of supratherapeutic levels in 87% of cases, significantly mitigating the incidence and severity of thrombocytopenia [10]. Notably, the rate of thrombocytopenia in patients with supratherapeutic levels whose doses were adjusted on the basis of TDM was comparable to those with desirable serum levels receiving standard dosing (10.3% vs. 10.7%).

A retrospective study conducted by Pea et al. involved 35 patients on linezolid monotherapy for longer than 28 days [11]. Linezolid TDM facilitated dose adjustments in 40% of patients to maintain serum concentrations below 10 mcg/mL. Among the 51.4% who developed thrombocytopenia, approximately one‐third achieved resolution through TDM‐guided dosing, underscoring its role in enhancing patient safety by tailoring therapy to individual pharmacokinetic profiles.

More recently, Laarhuis et al. reported a detailed case series and literature review highlighting the heightened risk of linezolid‐induced thrombocytopenia among patients with renal impairment, despite guideline‐recommended standard dosing [12]. Among five patients with reduced renal function, thrombocytopenia occurred after > 7 days of therapy, and was consistently associated with supratherapeutic linezolid trough concentrations, exceeding 10 mg/L. The authors suggest standard dosing in patients with eGFR < 60 mL/min/1.73 m^2^ confers a high probability of excessive exposure, whereas reduced dosing strategies of 50% substantially improve attainment of target trough concentrations (2–8 mg/L), and advocate for TDM 1 week after therapy initiation.

The findings of Lau et al. and Pea et al. affirm the broader applicability and benefits of TDM in reducing the discontinuation rates of linezolid due to toxicity. This is particularly relevant in patients like ours, where cessation of the only remaining effective antimicrobial agent due to toxicity development could lead to dire consequences. Moreover, our patient’s experience sheds light on the specific utility of TDM in long‐term suppression therapy in those most at risk for linezolid‐induced thrombocytopenia. Chronic suppressive therapy is often employed in patients with retained infected prosthetic material who have significant comorbidities or where surgical interventions (e.g., CIED removal or valve replacement) are not feasible. In these cases, the risk of drug toxicity assumes a greater significance due to the prolonged exposure period. TDM serves as a critical tool in optimizing patient‐specific dosing to ensuring both efficacy and safety over extended periods.

While TDM played a meaningful role in dose optimization in this case, its clinical effectiveness is inherently dependent on timely result availability. The prolonged turnaround time observed limited the ability to implement early dose adjustments, underscoring that TDM is most impactful when results are rapidly actionable. Streamlined workflows and reduced time‐to‐result are therefore essential for TDM to effectively mitigate linezolid‐associated toxicity, particularly in patients requiring prolonged therapy.

Ultimately, our patient’s clinical course supports the substantial value of TDM in the management of linezolid therapy for those at heightened risk of adverse effects, including those requiring long‐term treatment. This case acts as a compelling endorsement for the broader adoption of TDM practices, ensuring that linezolid’s efficacy is maximized and toxicities minimized at an individual patient level.

## Author Contributions

Kennedy C. Concannon and Taylor Gullickson contributed equally to the conception of the idea and drafting and revisions of the manuscript. Daniel C. DeSimone and Omar Abu Saleh contributed to revisions of the manuscript of oversight of the care of the patient. Tanner M. Johnson and Ryan W. Stevens contributed to drafting and revisions of the manuscript and care of the patient. Josh Clement contributed to the conception of the idea, revisions of the manuscript, and oversight of the writeup.

## Funding

No funding was received for this research.

## Consent

A waiver of informed consent was provided in accordance with MN Statute 144.295 allowing personal data processing from the patient.

## Conflicts of Interest

The authors declare no conflicts of interest.

## Data Availability

Data sharing is not applicable to this article as no datasets were generated or analyzed during the current study.
